# A Review of Research on the Neurocognition for Timbre Perception

**DOI:** 10.3389/fpsyg.2022.869475

**Published:** 2022-03-29

**Authors:** Yuyan Wei, Lin Gan, Xiangdong Huang

**Affiliations:** ^1^Department of Electrical and Information Engineering, Tianjin University, Tianjin, China; ^2^Department of Precision Instrument and Opto-Electronics Engineering, Tianjin University, Tianjin, China

**Keywords:** timbre perception, neurocognitive, psychology, EEG, ERP, fMRI

## Abstract

As one of the basic elements in acoustic events, timbre influences the brain collectively with other factors such as pitch and loudness. Research on timbre perception involve interdisciplinary fields, including physical acoustics, auditory psychology, neurocognitive science and music theory, etc. From the perspectives of psychology and physiology, this article summarizes the features and functions of timbre perception as well as their correlation, among which the multi-dimensional scaling modeling methods to define timbre are the focus; the neurocognition and perception of timbre (including sensitivity, adaptability, memory capability, etc.) are outlined; related experiment findings (by using EEG/ERP, fMRI, etc.) on the deeper level of timbre perception in terms of neural cognition are summarized. In the meantime, potential problems in the process of experiments on timbre perception and future possibilities are also discussed. Thought sorting out the existing research contents, methods and findings of timbre perception, this article aims to provide heuristic guidance for researchers in related fields of timbre perception psychology, physiology and neural mechanism. It is believed that the study of timbre perception will be essential in various fields in the future, including neuroaesthetics, psychological intervention, artistic creation, rehabilitation, etc.

## 1. Introduction

Timbre is a complex and abstract concept. Compared to other acoustic characteristics such as pitch and loudness, academic research on timbre started late and drew less attention, since timbre has been considered one of the most difficult acoustic features to comprehend. The Acoustical Society of America defined timbre in the 1960s as follows: the attribute of auditory sensation which enables a listener to judge that two nonidentical sounds, similarly presented and having the same loudness and pitch, are dissimilar. However, this definition only describes timbre from the dimension of loudness and pitch, rather than from the nature of timbre itself. In fact, timbre is not a single property, since it arises from an event produced by a single or several sound sources that are perceptually fused or blended into a single auditory image (Siedenburg et al., 2019). It contains not only auditory superficial features, but also rich auditory cognitive characteristics. Therefore, to systematically study timbre, it is necessary to integrate timbre perception with neurocognition, which requires a high-level interdisciplinary combination of psychology, physiology, neurology, physics, etc. To some extent, the difficulty of interdisciplinarity also leads to the fact that most of the timbre-relevant research works still stay in the exploratory stage.

Meanwhile, with the increasing progress of brain science and cognitive science, the last decade has witnessed an upsurge of interest in timbre. As far as research methods are concerned, besides the traditional behavioral science and psychology, the neural mechanism research based on brain imaging technologies such as EEG and fMRI has been increasingly applied. Regarding the spatial response of the brain stimulated by the timbre, the exploration range has extended from the initial auditory cortex to the overall analysis of multiple brain regions. Moreover, in the exploration of neural representation of timbre, besides peripheral auditory system, neurons researches at the mesoscopic level have also made further breakthroughs. For example, by imitating over 1,000 neurons in the mammalian primary auditory cortex as well as from simulated cortical neurons, Patil et al. (2012) constructed a neuro-computational framework to explore timbre classification. Meanwhile, the timbre stimuli also become increasingly complex, which have developed from simple mode of auditory stimuli (such as monophonic and synthetic sounds) to more complex stimuli with cognitive sensations (such as melody and natural sounds). These studies impose great significance on both the neural mechanism of the brain reaction to timbre and the aesthetic perception of timbre.

**Figure 1:**
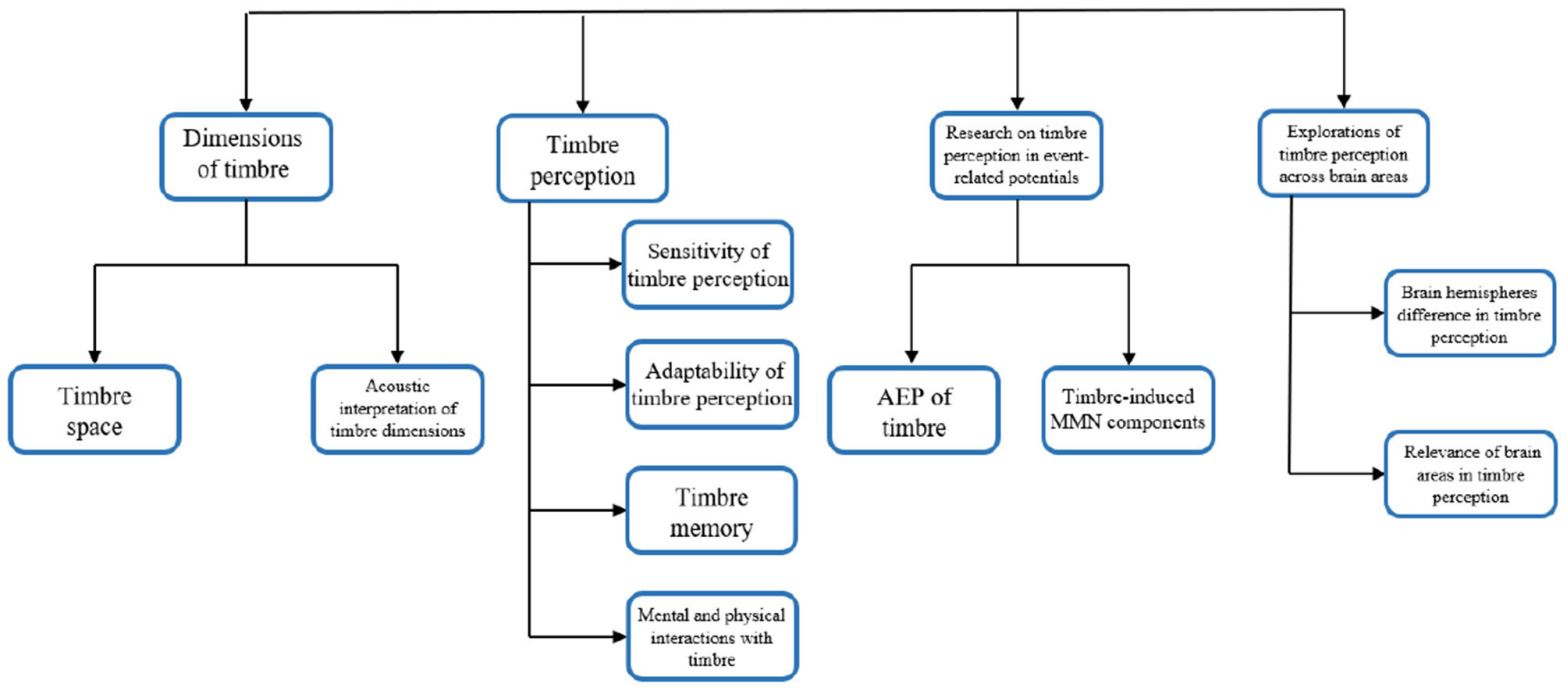
Overall structural diagram of this review.

This article aims to summarize the existing timbre-related research works in the field of neurocognition, which are sorted out into four parts. The first part addresses how researchers link the perceptual dimension of timbre to the quantitative dimension of acoustics from the perspective of psychophysics. The second part gives a comprehensive discussion on the brain's perception of timbre,which includes memory capability, adaptability etc. The third part focuses on the research of event-related potentials that are related to timbre. Finally, in the fourth part, the spatial distribution characteristics of brain perception on timbre are summarized. Besides, the overall diagram addressing the structural relationship of these four parts is illustrated in Figure 1.

## 2. Dimensions of Timbre

The earliest research of timbre can be traced back to the work of Helmholtz and Ellis (1855) and Stumpf (1926), and their research mostly studied timbre just from the perspective of physics which ignored the perceptual qualities of timbre. However, with the deepening of the timbre research, it has been found that timbre, as a complex perceptual property of a specific fused auditory event, is also involved in psychology and other disciplines. In the 1970s, a pioneering work was started by Plomp (1970) and Wessel (1973), who studied timbre perception from the perspective of the psychophysics. Following this, multiple dimensions were developed to study the psychological perception of timbre (Grey, 1977; McAdams, 1993; Handel, 1995; Hajda et al., 1997; Toiviainen et al., 1998). The following will describe in detail how these dimensions arise and what they refer to, from which we will also reveal some important but unsettled problems for discussion.

### 2.1. Timbre Space

**Figure 2:**
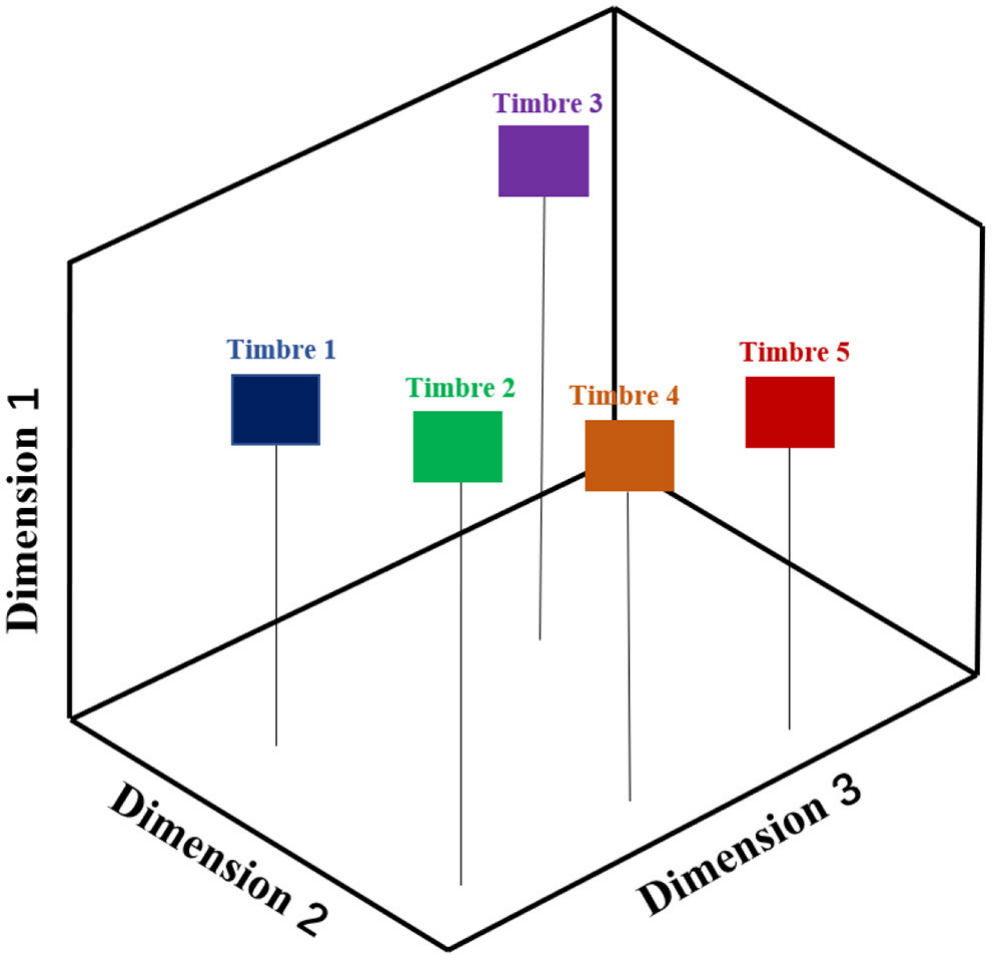
The three-dimensional timbre space diagram of five different timbres. The geometric distance between two timbres corresponds to the perceived differences between them, and the spatial dimensions are correlated with acoustical physical properties.

Through the exploration of the internal properties of timbre perception, the concept of timbre space was established through the well-known Multidimensional Scaling (MDS) research method (Grey, 1977; McAdams, 1993; Handel, 1995; Hajda et al., 1997; Toiviainen et al., 1998). This method links the people perception (psychology) to the timbre's physical properties (physics) *via* inference from the rating data of pairwise timbres, which can implement categorization of timbres without relying on any prior processing of the physical or perceptual structure of the timbre. Firstly, pairwise coupling is conducted among the given timbre set (i.e., any timbre should be transversely paired with other timbres). Secondly, all listeners are asked to rate the differences of all timbre pairs subjectively. Thirdly, these rating results are compared with each other so that a geometry space named “the timbre space” (an example of a three-dimensional timbre space diagram was illustrated in Figure 2) can be generated, from which the Multidimensional Scaling (MDS) model can be built up. In such timbre space, those timbres with similar properties tend to be closer to each other, and *vice versa*.

The basic MDS modeling method is based on the underlying assumption that all timbres are equally treated and all listeners are of the same level of perceptual ability (Kruskal, 1964a,b; Plomp, 1976). In other words, this modeling neither imposes any weight on certain special timbre nor makes any distinction among listeners. To further improve the modeling performance, a series of variants of MDS modeling approaches emerged by means of moderately relaxing this assumption. Among them, the EXSCAL algorithm (Krumhansl, 1989; Winsberg and Carroll, 1989) incorporates the specificity of every timbre. For the INDSCAL algorithm (Carroll and Chang, 1970; Wessel, 1973; Miller and Carterette, 1975; Plomp, 1976; Grey, 1977) and CLASCAL algorithm (Winsberg and Soete, 1993; McAdams et al., 1995), the listeners need to be categorized into several groups in terms of their abilities (or specificities) of timbre perception, which are treated with different weights accordingly. The CONSCAL algorithm (Winsberg and Soete, 1997; Caclin et al., 2005) can yield accurate models customized for individual listeners through continuous mapping operations on the timbre positions along perceptual dimensions by using spline functions. Generally speaking, since the above modified MDS algorithms can provide more accurate multidimensional timbre space, these variants tend to perform better in describing features, structures, and qualities of different timbres compared to the basic MDS method.

### 2.2. Acoustic Interpretation of Timbre Dimensions

The timbre space generated by the MDS modeling is about perception dissimilarity for sounds with similar pitch, duration, and loudness, and it represents the common perception dimension of timbre. A basic assumption is that these perceptual dimensions are orthogonal and should be represented by independent physical properties. These physical properties are used as an acoustic interpretation of timbre, which are called the audio descriptors.

These audio descriptors can be acquired by combining different perceptual dimensions and acoustic-related physical parameters, which can be categorized into descriptors of temporal, spectral, and spectrotemporal (Peeters et al., 2011). Temporal descriptions refer to the time aspect of sound. Some of them are directly extracted from waveforms, but most are usually extracted from time energy envelopes. Spectral descriptions usually refer to the local features of frequency contents. Spectrotemporal descriptions usually refer to the spectral changes across multiple time frames.

Generally speaking, most studies (Grey and Gordon, 1978; Iverson and Krumhansl, 1993; Krimphoff et al., 1994; McAdams et al., 1995; Kendall et al., 1999) agree that the following descriptors can represent the characteristics of different timbres: (1) Spectral centroid: It represents the relationship between low and high harmonics. Specifically, the greater the amplitudes of the high- frequency components relative to the low-frequency components are, the higher the spectral centroid is and thus the clearer and brighter the sound is. For example, the oboe has a higher spectral centroid than the French horn. (2) Attack time: It indicates a transition period, during which the amplitude of a particular harmonic increases from the perceptible threshold level to the maximum value. The shorter the attack time is, the more acute the timbre feels. For example, string instruments have a longer attack time than percussion instruments. (3) Spectral flux: The evolving degree of the spectral shape within a duration. (4) Spectral irregularity: It is relevant to the intensity of even harmonics relative to odd harmonics. If the amplitudes of even harmonics are relatively lower than odd harmonics, the sound tends feel hollow.

Audio descriptors play important roles in characterizing the psychoacoustics of timbre, which help explain the timbre perception in acoustic fields. However, the current research on timbre descriptors is still in confusion: how many descriptors can comprehensively describe a timbre? How does people perceive timbre?—with a linear or nonlinear combination of descriptors? How to evaluate the interpretability of an individual descriptor? These questions are still worth further exploration.

## 3. Timbre Perception

Limited by the multidimensional and complex characteristics of timbre, either the traditional research based on acoustic characteristics or the spectrum analysis and the psychological subjective evaluation meets challenges in exploring timbre perceptions. Since timbre perception is ultimately fulfilled by the brain, some studies attempted to combine physical cognition and neural perception of timbre for the purpose of uncovering the interactions between different timbre dimensions. For example, Caclin et al. (2006) showed that different dimensions of timbre are processed in parallel when the brain perceives timbre. In conclusion, it is an indispensable work to explore the mechanism on how the brain processes timbre perception.

### 3.1. Sensitivity of Timbre Perception

Studies have found that the human brain can perceive the difference of timbre. Peynirciou et al. (2016) attempted to acquire timbre perception difference through the artificial mixture of real musical instruments. They conducted two experiments. In one experiment, the subjects were asked to hear several fragments of the mixture of different instruments, from which they judged the mixing degree of these musical instruments. In the other experiment, the participants were asked to identify the different timbres that contained specific proportion of mixed instruments. These two experimental results showed that participants could accurately perceive the timbre differences of these instruments. Moreover, it was also found that the subjects who received music training and those who did not receive music training showed similar response patterns. Meanwhile, Samson et al. (1997) conducted experiments using synthesized timbres, which only subtly differed in frequency spectra and time information. They proved that the human brain was very sensitive to the perception of timbre differences, which was consistent to the conclusion drawn by Peynircioǧlu.

### 3.2. Adaptability of Timbre Perception

The human brain has a certain degree of adaptability to the perception of timbre (i.e., the perceptual after-effect). To verify this effect, Piazza et al. (2018) asked the participants to be repeatedly exposed to two sounds (e.g., clarinet and oboe, male and female voice) and then these subjects were asked to hear one of them. The results showed that: when the subjects solely listened to sound A (or B), they naturally incorporated the auditory perception effects of A with those of B. Moreover, the experiment also proved that such after-effects were robust for moderate pitch changes. This adaptation contributes to the stability of timbre perception and the extensibility of familiar timbre. It actually enhances the sensitivity to novel or rare auditory objects, such as the timbre of an unfamiliar human voice.

### 3.3. Timbre Memory

It is often taken for granted that timbre can be easily memorized in the brain. However, the memory of timbre actually requires a complex mnemonic architecture, which delicately keeps track of sound identities and concurrently manages timbre operations (such as sensory processing, information storage, and matching of representations). Poulin-Charronnat et al. (2004) found that changing the instrumental timbre will affect the memory of tonal excerpts in human brain during the study of tonal and atonal music memory. Trainor et al. (2004) also found that the timbre change perturbs infants' melody memory. In the study on synthesized timbres, Golubock and Janata (2013) found that all the differences along the dimensions of spectral centroid, attack time, and spectral flux would influence the capacity of working memory. Meanwhile, Schellenberg and Habashi (2015) studied the memory capability influenced by the lags of timbre stimuli. Specifically, in the melody recognition test, by altering the lags between exposure and test spanning, which were set as 10 min, 1 day, and 1 week, they surprisingly discovered that the lag alternations did not significantly affect the timbre memory capability.

### 3.4. Mental and Physical Interactions With Timbre

In some circumstances, changing in timbre may awaken people's overall physiological and psychological responses, which is especially obvious in sensorimotor system. Many studies (Behrens and Green, 1993; Gabrielsson and Juslin, 1996; Leman et al., 2009) have found that musicians can use gesture language to convey emotional intentions under the influence of timbre. Therefore, people may not just passively listen to different timbres, which means, timbre changes in turn can also promote the extent of musicians' involvement in the overall state of the body (Halpern et al., 2004). Overy and Molnar-Szakacs (2009) proposed the “shared affective motion experience” (SAME) hypothesis based on the basic level of noisy and normal timbres. Combined with behavioral research, Blumstein et al. (2012) proved that noisy timbre could cause more vigorous physical activity than non-noisy timbre in terms of evoking the limbic nervous system response. Following this, Wallmark et al. (2018) supplemented the consensus of the predecessors through the embodied cognition research paradigm. They conducted experiments on different monophonic timbres and composite music timbres, which were then converted into noisy timbres by means of pitch shifting techniques. Their experimental results show that, such noisy timbres are able to arouse greater physical exertion and produce a lower emotional response than a non-noisy timbre, and that the noisy timbres can evoke responses in the motor system of the brain. It can be seen that, in addition to the significant impact on the human brain's auditory dimensions, timbre can also inspire a listener to produce emotional actions, which in turn reflects the perception of the listener.

Although efforts have been extensively made to study the brain responses of timbre, there are still many unsolved problems including the brain perception process of timbre identification, the exploration of brain regions for different functionalities of timbre perceptions etc. Therefore, the field of timbre perception study is still very young, which is expected to bring about breakthroughs through integrating varieties of neurocognitive experimental methods and new techniques of data processing such as EEG/ERP and fMRI.

## 4. Research on Timbre Perception in Event-Related Potentials

As a popular physiological means to effectively reflect human brain activities, EEG (electroencephalogram) has been increasingly used in the research of brain cognitive mechanism. The EEG arises from the potential oscillations in the brain (i.e., excitatory postsynaptic potentials), and the current is afferent from the cortex of the thalamus to activate the parietal dendrites (Schaefer, 2017). Early EEG experiments mainly focused on the oscillation of the brain wave (which refers to spontaneous EEG), whose voltage can be collected in the experimental process. In contrast, later studies paid more attention to a special EEG component that had a time-locked relationship with psychological events, namely ERP (event-related potential). ERP has three notable features: one is that the waveform change is either positive or negative; the second is that the waveform change should behave as a sufficiently high intensity (amplitude); and the third is that the wave change occurs at a specific moment after the stimulus (latency period) are triggered (Wang, 2011). Because ERP can reflect mental activity in millisecond accuracy and thus has a high time resolution without causing any brain damage, it is applicable to explore the brain cognitive mechanism of short-term sound stimulus, such as the brain response to transient timbre stimulation.

### 4.1. AEP (Auditory Evoked Potential) of Timbre

Early studies have found that for auditory stimuli, N1 (Näätänen and Picton, 1987) and P2 (Celesia, 1976) are typical ERP components that reflect human auditory perception and auditory classification. Therefore, in numerous electrophysiological studies (Auzou et al., 1995; Liu et al., 2018; Hamlin et al., 2019; Banerjee et al., 2021) of timbre perception, researchers often treat N1 and P2 as indicators to reveal the neural mechanism of timbre processing.

Many studies (Helmholtz and Ellis, 1855; Fletcher, 1934; Seashore, 2008) have found that the timbre is closely related to the harmonic structure. Therefore, some researchers have carried out investigations on pure tone (lacking harmonic structures) and complex tone with the same baseband but different harmonics. Meyer et al. (2006) obtained ERP from 16 healthy subjects, who were required to distinguish between complex instrumental monophonic sounds (piano, trumpet, and violin) and simple pure sounds that lacked timbre characteristics. Analyses showed that, compared to pure tones, N1 and P2 responded more strongly to the tones of the instrument. At the same time, Tardón et al. (2021) attempted to discover the variations of the electrophysiological responses of the brain by simultaneously changing the acoustic characteristics of music, demonstrating that the amplitudes of the N1 and P2 components increased when the spectral flux, one of the dimensions of timbre, was mutated. Moreover, Pantev et al. (2001) observed that, for professional trumpet players and violinists, the timbre arising from playing their own instruments tended to evoke stronger N1 event-related potential components than other timbre did.

Besides the timbre stimuli, AEP can also be evoked *via* auditory imagination of timbre. Studies (Tuznik et al., 2018) have proved that the difference in imaginary timbre can be reflected in event-related potentials. Specifically, they found that timbre imagination is able to evoke other ERPs such as LPC in addition to N1 and P2. Moreover, LPC was found more sensitive to timbre changes of imagined sounds than other AEPs. In addition, it was also discovered that, once the ERP was successfully evoked, whether a subject had experienced music training or not, the magnitude of N1, P2, and LPC potentials were not affected. Nevertheless, when performing the same auditory imagination tasks related to timbre, the success rate of musicians was higher than that of non-musicians.

### 4.2. Timbre-Induced MMN Components

MMN (Mismatch Negativity) is an important component of event-related potentials (Luck, 2009), which is obtained by the Oddball paradigm. This paradigm involves two types of sound stimuli: standard stimuli and deviation stimuli. The standard stimuli appear with high probability, whereas deviation stimuli appear with low probability. To acquire MMN, ERPs evoked by both the standard stimuli and the deviation stimuli is need to be respectively superimposed and averaged. Then, the ERP evoked by the deviation stimuli is subtracted from the ERP evoked by the standard stimuli, from which a difference wave can be generated and treated as the desired MMN. The waveform of a MMN appears as a negative deflection, which occurs during 100–250 ms after the stimulus (i.e., the latency period is 100–250 ms; Näätänen et al., 2004). Particularly, the MMN can also be evoked even if the listener is in the coma state, which can be applied as an automatic indicator of the hearing mechanism recovery in the early treatment on the hearing-impaired.

Christmann et al. (2014) explored how timbre variation affected the MMN on the condition that other variables remained unchanged by means of the spectrally rotated technique. The experiment proved that, MMN evoked by instrumental sounds with timbre characteristics occurred earlier than those evoked by pure tones without timbre characteristics. This result indicates that the brain tends to be more sensitive to tones with rich harmonic structures, but is not influenced by pitch changes. Caclin et al. (2008) measured the variations of MMN by altering single-dimensional timbre characteristics (attack time, spectral centroid, even harmonic attenuation) and their combinations, from which he concluded that there existed some neural cells dedicatedly processing acoustics in the brain. Torppa et al. (2018) found that when the children with cochlear implants (CIs) perceived timbre differences (from piano to Cymbal) in noise, the amplitude of MMN would change significantly. This conclusion suggests the importance of MMN in studying the timbre perception of children with CIs in noisy circumstances.

Since MMN is sensitive to varieties of timbre characteristics, it is usually employed as an indicator for timbre classification. Specifically, by altering the types of standard timbre stimuli or deviation timbre stimuli, MMN of different features (such as amplitudes, latency periods) can be evoked, thus reflecting listeners' abilities in identifying varieties of timbres, such as distinguishing between pure tones and overtones (Tervaniemi et al., 1997), distinguishing timbres with different spectral complexities (Tervaniemi et al., 2000), and distinguishing musical timbres that convey different emotions (Goydke et al., 2004). In addition, concerning applications of MMN, it was found that people with cochlear implants still had the ability to distinguish timbres although they were weaker than normal people (Koelsch et al., 2004).

In general, various ERP components induced by timbre actually provide a window to observe brain responses to timbre changes, which also helps researchers explore the neural mechanism of the brain's timbre perception. Nevertheless, to further discover this mechanism, it is necessary to analyze the timbre response distinctions across different brain areas.

## 5. Explorations of Timbre Perception Across Brain Areas

With the development of magnetic resonance imaging technology, fMRI (functional Magnetic Resonance Image) and PET (Positron Emission Computed Tomography) have gradually been adopted, through which the response mechanism of the brain to timbre perception in different spatial locations is being discovered. Related studies cover the spatial characteristics of brain's perception on spectral and temporal information of sounds (Zatorre and Belin, 2001; Hall et al., 2002), the effect on the spatial location distribution of brain from variations of timbre harmonics (Menon et al., 2002), the response difference of brain locations to the sound spectrum envelope (Warren et al., 2005), etc. These studies related to spatial features will facilitate the extension of explorable brain regions for timbre perception, such as the areas spreading from the auditory cortex to the whole brain.

### 5.1. Brain Hemispheres Difference in Timbre Perception

Some studies based on auditory perception have found that the left brain has an advantage in the processing of sound properties in the time domain (Robin et al., 1990), while the right brain has an advantage in dealing frequency-domain information (Zatorre and Belin, 2001; Menon et al., 2002). And based on the disclosure that timbre is closely related to both harmonic structure and time structure, researchers have found that timbre discrimination relies on the whole auditory cortex in the brain, while at the same time timbre perception also has the right-side advantage (Platel et al., 1997).

Studies have proved that the temporal lobe is involved in the brain's processing of timbre. On this basis, Samson and Zatorre (1994) carried out the timbre discrimination study on patients with unilateral temporal lobe resection, and found that only those subjects with right temporal lobe resection were affected in timbre discrimination. These findings supported the functional role of the right temporal lobe in timbre discrimination, and the responses of those subjects with left temporal lobe resection were not obvious. Leaver and Rauschecker (2010) also proved that right superior temporal regions were active in the processing of different timbres on instruments. However, their conclusion that the basic attributes of music perception were mainly biased toward the right hemisphere was then challenged by Johnsrude et al. (1997). Specifically, their study examined the processing of attack time in non-percussion sounds by using PET technology, which showed that the subjects had obvious activation foci in the left orbitofrontal cortex and left fusiform gyrus. Later, Samson et al. (2002) also showed that both the left and right hemispheres were involved in timbre processing. They found that the patients with left temporal lobe lesions were not influenced in distinguishing single sounds, but when the single sounds appeared in the background of a melody, the patients were unable to judge the degree of dissimilarity. At the same time, Menon et al. (2002) also revealed that the left brain and right brain exhibited some asymmetry in reaction to timbre stimuli. Such asymmetry expressed in the fact that the activation of the left temporal lobe was significantly posterior compared with the right hemisphere.

### 5.2. Relevance of Brain Areas in Timbre Perception

At present, studies have shown that the response of cerebral cortex to sound stimulus is mainly distributed in areas of the primary auditory cortex, superior temporal gyrus, superior temporal sulcus and Heschl's gyrus, prefrontal ventrolateral area, etc. (Platel et al., 1997; Caclin et al., 2007; Samson et al., 2011; Wallmark et al., 2018). Beside the above areas, some researchers believe that timbre, as a complex multi-dimensional perceptual property, may be related to the activities of brain areas that do not correspond to the processing of some low-level auditory stimuli. Thus, Meyer et al. (2006) used the EEG imaging method of low-resolution electromagnetic tomography (LORETA) and proved that timbre perception involved not only the two sides of the auditory cortex, but also the middle region of the brain that was related to emotion and auditory imagination. The research of Alluri et al. (2012) proved that when participants listened to timbres of “bright” qualities, the putamen (basal ganglia) would be activated. Wallmark et al. (2018) explored the neural dynamics of single-tone timbres at different noise levels to determine which areas of the brain were involved in the processing of noisy brain stimuli. It turned out that timbre processing was related to the sensorimotor area. At the same time, Blumstein et al. (2012) confirmed that the motion and edge responses caused by different timbres had certain differences.

The brainstem, an important part of the central auditory system, has also been studied to explore timbre perception. Strait et al. (2012) revealed that, a musician's auditory brainstem behaved as unique responses to his own frequently-exercising instrument, whereas it also showed insensitivity to other instruments with distinct timbres. By reviewing the work on the auditory brainstem's ability to respond to complex sounds, Anderson and Kraus (2010) found that timbre can be applied as an objective neural index for hearing-in-noise abilities.

## 6. Discussion

On basis of the above retrospect, the findings of timbre perception can be obtained by either psychological approaches or physiological approaches. The psychological approaches typically refer to the basic multi-dimensional scaling modeling method and its variants, from which a series of audio descriptors concerning the related dimensions can be derived. The physiological approaches usually rely on signal acquisition means that are related to cerebral neural activities including EEG/ERP, fMRI, and PET, which provide various perspectives to explore the neural mechanism of the brain's timbre perception.

In general, it can be concluded that timbre perception is promising in psychology- and neurocognition-related fields. Specifically, timbre perception can play crucial roles in future applications such as music creation, auditory neuroaesthetics, and human-computer interaction experiences, if efforts are made in the following directions.

(1) Interdisciplinary fusion should be strengthened. Up to now, in both the timbre space modeling and timbre abstract encoding from low to high levels in the brain, the inter-discipline permeation is still not sufficient. For example, the aforementioned mental and physical interaction with timbre stimuli can hardly be interpreted well due to the lack of inter-discipline permeation. Therefore, it is urgent to integrate multiple academic fields including psychology, neurocognition, physical acoustics etc. Only in this way can the mechanism of the timbre-relevant perception be explored deeply.

(2) More attention should be paid to the relevance with other acoustic characteristics. Most of the existing timbre research only focuses on the characteristics of timbre itself. In fact, because timbre rarely appears in a single form, timbre perception inevitably is affected by other characteristics such as pitch, loudness, melody, and rhythm. Therefore, it is necessary to incorporate the relevance between timbre and other characteristics. Essentially,if the timbre research is placed in a comprehensive environment which organically links all these elements, the study of timbre perception will be more productive.

(3) More emphasis should be placed on individual differences. At present, only few studies address individual differences in timbre perception, and most of them only focus on the difference between musicians and non-musicians. Nevertheless, higher diversity should be considered concerning individual difference. For example, individuals with hearing impairments or with poor auditory perception should also be considered. The efforts on exploring the characteristics of special individuals' timbre perception will further promote the advancement of auditory aesthetics and neuromedicine.

## Author Contributions

YW: data collection, data analyses, and writing the article. LG: study idea, study design, and manuscript revision. XH: study design and manuscript revision. All authors contributed to the article and approved the submitted version.

## Funding

This study was supported by a grant from National Natural Science Foundation of China (No. 6210-7029).

## Conflict of Interest

The authors declare that the research was conducted in the absence of any commercial or financial relationships that could be construed as a potential conflict of interest.

## Publisher's Note

All claims expressed in this article are solely those of the authors and do not necessarily represent those of their affiliated organizations, or those of the publisher, the editors and the reviewers. Any product that may be evaluated in this article, or claim that may be made by its manufacturer, is not guaranteed or endorsed by the publisher.
